# Oncolytic viruses for triple negative breast cancer and beyond

**DOI:** 10.1186/s40364-021-00318-4

**Published:** 2021-09-25

**Authors:** Shengye Jin, Qin Wang, Hao Wu, Da Pang, Shouping Xu

**Affiliations:** 1 Harbin Medical University, 157 Baojian Road, Harbin, 150086 China; 2 Department of Breast Surgery, Harbin Medical University Cancer Hospital, 150 Haping Road, Harbin, 150081 China; 3 Sino-Russian Medical Research Cen8ter, Harbin Medical University Cancer Hospital, 150 Haping Road, Harbin, 150081 China; 4 Heilongjiang Academy of Medical Sciences, 157 Baojian Road, Harbin, 150086 China; 5 Translational Medicine Research and Cooperation Center of Northern China, Harbin Medical University, 150 Haping Road, Harbin, 1550081 China

**Keywords:** Oncolytic virus, Triple negative breast cancer, Immune checkpoint inhibitor, Chimeric antigen receptor T cell, Combination therapy

## Abstract

Biological therapy is considered an alternative treatment capable of eliciting the same effects on tumors as surgery, radiotherapy, and chemotherapy. As a major player in biological therapy, oncolytic viruses (OVs) have attracted great attention and achieved good results. Specifically, the successful application of OVs in head and neck cancer, as well as melanoma, promoted its research in triple negative breast cancer (TNBC). TNBC is a high-risk molecular type of breast cancer, characterized by strong invasion, easy recurrence, and metastasis. Due to the absence of estrogen and progesterone receptors, as well as the absence of overexpression or gene amplification of human epidermal growth factor receptor 2 (HER2), endocrine therapy and anti HER-2 targeted therapy have proven ineffective. Although chemotherapy has shown substantial efficacy in some TNBC patients, the occurrence of drug resistance and poor prognosis have prompted the exploration of new and effective treatment methods. The emerging concept of OVs provides a new platform to treat TNBC. Indeed, several studies have confirmed the therapeutic effects of OVs in TNBC. Numerous studies have also investigated the efficacy of OVs in other malignances, including solid tumor clinical trials, thus further demonstrating the promising application of oncolytic virotherapy for TNBC. The primary focus of the current review is the examination of OV mechanisms underlying their antitumor properties, while also summarizing the ongoing progress in OV research regarding TNBC treatment, as well as the various combinatorial strategies comprising OVs and other therapies. We also briefly introduce specific relevant clinical trials and discuss some of the progress in the research of novel OVs for the treatment of other malignancies, thereby affirming the significant therapeutic potential of OVs for the treatment of TNBC, as well as other cancers.

## Background

Oncolytic virotherapy is a new biological treatment method under extensive research. Oncolytic viruses (OVs) are natural or artificially modified viruses that selectively infect and destroy tumor cells, rarely damaging normal cells. The tendency of OVs to infect tumor cells is attributed to the specific cytokines expressed by malignant cells and damaged signal pathways that lead to the destruction of cellular antiviral defense, thereby making tumor cells particularly susceptible to OVs [1, 2]. OVs were first discovered in patients with leukemia, whose symptoms improved after influenza infection [3]. Later, Italian doctors found that rabies vaccine could inhibit cervical cancer. This phenomenon led scientists to postulate that certain viruses have a tumor-resistant function. In subsequent studies, many viruses with the ability to kill tumor cells were discovered, leading to the emerging concept of OV therapy. Initially, studies focused on the antitumor effect of wild type viruses. A clinical trial in the 1950s used a wild type and non-engineered adenoidal pharyngeal conjunctival virus to treat cervical cancer [4]. With the development of genetic engineering, recombinant selectivity-enhanced viruses and therapeutic transgene-delivering ‘armed’ OVs, were widely studied [5–7]. Based on the nucleic acid types, the most widely studied OVs can be divided into double-stranded (ds) DNA viruses [adenovirus, herpes simplex virus (HSV), and vaccinia virus], dsRNA viruses [reovirus], positive-sense single-stranded (ss) RNA viruses [coxsackievirus], and negative-sense ssRNA viruses [vesicular stomatitis virus (VSV), measles virus (MV), Maraba virus, and Newcastle disease virus (NDV) [8].

Breast cancer has a high prevalence rate and the highest mortality rate in women [9]. Breast cancer can be divided into the molecular types—luminal A, luminal B, ERBB2+, and triple negative breast cancer (TNBC)—based on different expression profiles of the estrogen receptor (ER), progesterone receptor (PR), and human epidermal growth factor receptor 2 (HER2) [10]. TNBC is found in approximately 15% of breast cancer patients and is characterized by no expression of ER and PR as well as no amplification of the HER2 gene [11, 12]. TNBC has a more aggressive clinical course, greater tendency to metastasize to other organs, higher risk of distant recurrence, earlier recurrence, and poor prognosis than other forms of breast cancer [13–15]. Currently, commonly used clinical treatment methods, such as surgery, radiotherapy, and chemotherapy, have not achieved a satisfactory outcome for this cancer subtype. Although a better pathologic complete response rate can be achieved by neoadjuvant chemotherapy, overall prognosis remains poor [16]. Hence, new targets of TNBC have been studied, however, no success has yet been reported in clinical practice. Currently, the treatment approach primarily relies on conventional chemotherapy [17]. However, disease progression, metastasis, or recurrence occurs due to the occurrence of chemotherapeutic drug resistance. Specifically, P-glycoprotein (P-gp), multidrug-resistant protein-1 (MRP1), and breast cancer resistance protein (BCRP) contribute to the development of chemotherapeutic resistance by increasing drug efflux and, subsequently, TNBC metabolism, leading to the replacement of the chemotherapeutic agents cannot benefit patients more [18]. Hence, an urgent need exists for the development of novel treatment strategies for TNBC. Oncolytic virotherapy may represent one such strategy for patients presenting with chemotherapeutic drug resistance. Furthermore, combination oncolytic viral therapy may be effective for TNBC patients who have undergone immunotherapy with poor outcomes as this strategy facilitates activation of the immune response via OV infection. Additionally, TNBC patients with poor physical conditions who are unable to tolerate surgical radiotherapy or chemotherapy may be candidates for oncolytic virotherapy as these viruses have been shown to elicit minimal impairment of healthy bystander cells with few adverse side effects.

Currently, a few OVs have been approved for clinical application. Oncorine (H101), a recombinant oncolytic adenovirus was approved for the treatment of head and neck cancer by the China Food and Drug Administration in 2005 [19]. Talimogenelaherparepvec (T-VEC), an oncolytic HSV type-1, was approved by the United States Food and Drug Administration for the treatment of melanoma lesions in the skin and lymph nodes in 2015 [20]. Hence, given the well-established therapeutic efficacy of OVs in malignant tumors, they represent a therapeutic approach with significant potential for the treatment of solid tumors, including those associated with breast cancer. The present review highlights the antitumor mechanisms of OVs and focuses primarily on research progress regarding OVs in TNBC. It further assesses the feasibility and research status of oncolytic virotherapy alone and in combination with other therapies. Finally, an introduction to clinical trials examining the efficacy of novel OVs in other malignant tumors, is provided to further demonstrate the potential of OVs for the treatment of TNBC.

### Antitumor mechanisms of OVs

**Fig. 1:**
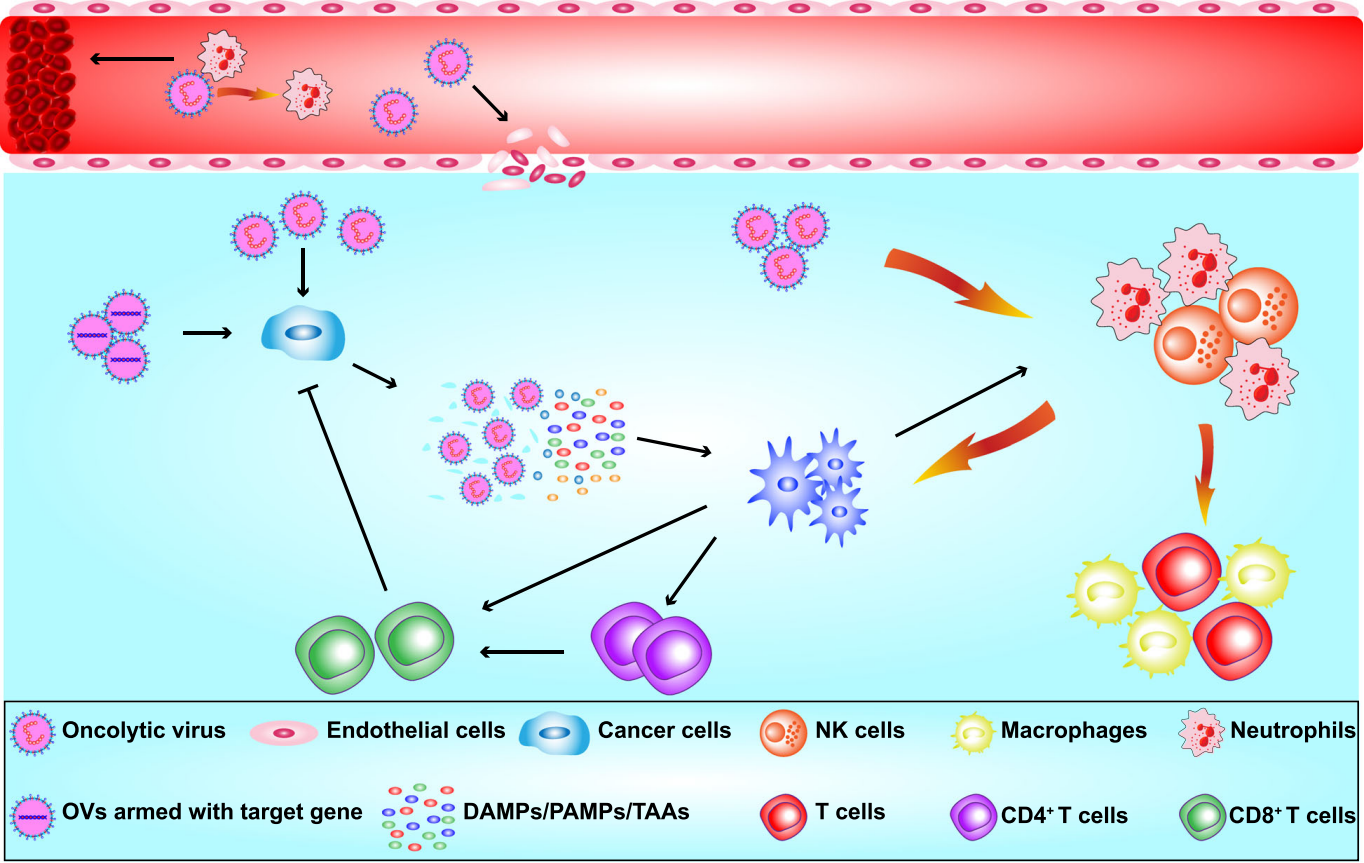
Underlying mechanisms responsible for the antitumor effects of oncolytic viruses (OVs). (1) OVs can directly infect and lyse cancer cells. (2) OVs infect and lyse vascular endothelial cells, and recruit neutrophils, which promote coagulation and thrombosis to destroy blood vessels and inhibit angiogenesis. (3) OVs recruit dendritic cells (DCs), macrophages, neutrophils and T cells into the tumor microenvironment thus transforming “cold” tumors into “hot” tumors. (4) Lysed cancer cells release DAMPs/PAMPs/TAAs, which are recognized by DCs resulting in activation of CD4 + T and CD8 + T cells to kill cancer cells. (5) OVs can be armed with transgene target gene expression, which enhances the antitumor effect

Initially, the understanding of the antitumor effect of OVs was limited. They were considered to replicate and directly lyse the tumor cells after infecting them, a process termed oncolysis. However, further research revealed that the antitumor effect of OVs is the result of multiple mechanisms (Fig. 1), which are reviewed here.

#### Effect of OVs on vasculature

Tumor growth requires a large supply of nutrients and oxygen as well as the excretory system for excreting metabolites generated due to the infinite proliferative character of tumor cells. Host vasculature is inadequate to meet these demands; thus, angiogenesis is initiated. Considering the role of angiogenesis in tumors, its inhibitors, such as Bevacizumab, were developed for cancer treatment. OVs were revealed to play an antitumor role by affecting tumor vessels. Studies revealed that some OVs destroy tumor vasculature via the infection and direct lysis of tumor-associated endothelial cells (ECs) as well as lead tumor cells to inflammatory responses and release of tumor necrosis factor (TNF)-α and interferon (IFN)-γ [21]. In addition, OVs can reduce the expression of vascular endothelial growth factor (VEGF), thereby inhibiting angiogenesis [22]. Benencia et al. demonstrated that mutant HSV-1716 virus (double g34.5 negative) infects tumor endothelium, and causes destruction of tumor vasculature both in vitro and in vivo [23]. In addition, OV-induced influx of neutrophils can lead to coagulation and loss of tumor perfusion [24, 25]. Furthermore, a natural tumor vasculature targeting capacity has been reported for VSVs, leading tumor blood vessel coagulation and, ultimately, vascular collapse [25, 26]. Thus, OVs play an antitumor role by affecting tumor vessels.

#### Interaction between OVs and immune system

Another remarkable antitumor mechanism of OVs is their induction of immune response. OV infection recruits innate immune cells, such as natural killer (NK) cells and neutrophils that form a defense system for virus elimination. Meanwhile, some of these cells also produce an antitumor effect. For instance, NK cells kill malignant cells by releasing cytotoxic granules, such as perforin and granzymes, as well as by secreting death receptor ligands (FasL and TNF-related apoptosis inducing ligand (TRAIL)) and cytokines, such as TNF-α, granulocyte-macrophage-colony-stimulating factor (GM-CSF), and IFN-γ [27, 28]. Additionally, neutrophils were shown to cause tumor cell death via TNF-α expression that led to localized acute ischemia due to perfusion loss [26, 29]. Simultaneously, OV-induced tumor cell lysis results in the release of viral pathogen-associated molecular patterns (PAMPs), danger-associated molecular patterns (DAMPs), and tumor-associated antigens (TAAs), which play a vital role in activating antitumor immune response. PAMPs, comprising virus surface structures such as capsids, viral genetic material, and metabolites generated during viral replication, are recognized by pattern recognition receptors (PRRs), such as toll-like receptors (TLRs), IFN-inducible dsRNA-activated protein kinase, and NOD-like receptors, that are expressed on dendritic cells (DCs) [30]. The interaction between PRRs and PAMPs stimulates DCs to produce cytokines such as TNF-α, interleukin (IL)-12, and type-1 IFNs (IFN-α and IFN-β) [31], which in turn promotes DC maturation to further release proinflammatory cytokines, thereby activating and recruiting more NK cells or DCs and, thus, amplifying the antitumor immune response. DAMPs, including ATP, calreticulin (CRT), and high mobility group protein B1 (HMGB1), are secreted or released by dying, stressed, or injured cells [32]. ATP, CRT, and HMGB1 bind to P2RX7, CD91, and TLR4, respectively. ATP can stimulate the recruitment of DCs to cancer cells, CRT enhances the engulfment of tumor antigens by DCs, and HMGB1 promotes antigen presentation to T cells [33]. Subsequently, DCs expose TAAs via MHC-I molecules to T cell receptors on cytotoxic CD8+ T cells (cytotoxic T lymphocytes, CTLs) to induce the differentiation of naïve CTLs to effector CTLs. The antitumor activity of CTLs is achieved either directly by releasing cytotoxic particles containing perforin and granzyme or indirectly by secreting cytokines such as IFN-γ, TNF-α, and IL-2 [34, 35]. These cytokines induce tumor cell apoptosis and/or activate anticancer immune response [36], which serves as a bridge between the effects of OV on innate immunity and adaptive immunity.

#### OVs ‘heat up’ tumors

In the past 5 years, the novel immune-based concept of ‘hot’ and ‘cold’ tumors has been proposed. Tumors are classified based on the distribution of immune cells within the tumor site by determining immune cell infiltration and Immunoscore ranges from I0 (for low immune cell densities with the absence of both cell types in both regions) to I4 (high immune cell densities in both locations). Tumors with non-infiltrates corresponding to Immunoscore I0 are designated as ‘cold’ tumors, while highly infiltrated tumors corresponding to Immunoscore I4 are called ‘hot’ tumors [37]. Due to the lack of immune cells in the tumor microenvironment (TME), ‘cold’ tumors are not sensitive to immune response, a characteristic that is responsible for the unsatisfactory effect of several treatment methods in these types of tumors. However, OVs can override this condition. That is, following OV infection, the immune response first recruits neutrophils and macrophages to eliminate the OVs and infected cells. Meanwhile, DCs sense viral PAMPs and produce type I IFNs capable of directly activating NK cells, which subsequently produce IFN-γ and TNF-α to further activate and recruit macrophages, DCs and T cells [2]. Since OVs have a greater propensity to infect tumor cells, these responses ultimately result in the infiltration of immune cells to the tumor microenvironment (TME). Thus, ‘cold’ tumors become ‘heated,’ enhancing the antitumor immune response.

#### OVs carry target gene

Viruses can carry foreign genes, replicate in the host, and are readily modified in vitro. As such, viruses are often used as vectors for genetic engineering, the rapid development of which has facilitated the application of OVs for the induction of antitumor effect by arming them with target genes that either encode antitumor cytokines or promote antitumor immune responses [24, 38, 39]. The success of T-VEC, a GM-CSF-armed oncolytic HSV, has clearly demonstrated feasibility of this method. Furthermore, a recombinant VSV expressing IFN-β was reported to exhibit oncolytic activity against lung cancer in vivo and in vitro [40].

In summary, OVs represent potential candidates for the treatment of cancers owing to their abilities to replicate in tumor cells causing their direct lysis, destroy tumor vasculature, activate innate and adaptive immune responses to generate an antitumor effect, converting ‘cold’ tumors to ‘hot’ ones, and function as a vector, carrying target genes and expressing antitumor related factors in the tumor cells. These characteristics and antitumor mechanisms make OVs advantageous compared to other treatment methods, including radiotherapy, chemotherapy, and endocrine therapy. Moreover, their preference for tumor cells makes OVs highly effective, with fewer adverse events and drug resistance. Further, since viruses actively replicate themselves after entering host cells, causing subsequent activation of the immune system, oncolytic virotherapy is also capable of maintaining long-term antitumor effects.

### OVs in TNBC

TNBC, a high-risk breast cancer subtype, has limited treatment options and, thus, may benefit from oncolytic virotherapy. Indeed, several studies on the application of oncolytic virotherapy in TNBC have demonstrated the feasibility of this treatment strategy. Here, we introduce different OVs that have been studied in TNBC. In addition, we summarize OVs that have reportedly achieved good results in preclinical studies (Table 1) [41–60].

**Table 1:** Summary of OVs against TNBC

Virus	Receptor	Vector	Modification	Mechanism	Ref
Adenovirus	Coxsackie and adenovirus receptor (CAR), CD46, Desmoglein-2(DSG-2)	CNHK600-IL24	Arm with IL-24	Induce TNBC apoptosis	[41]
		p55-hTERT-HRE-TRAIL	Arm with P55-HTERT-HRE-TRAIL	Target TNBC and induce apoptosis	[42]
		OBP-401 adenovirus	Arm with the human telomerase reverse transcriptase *(hTERT)* gene	Increase the targeting to cancer cells	[43]
		Ad5-10miR145T	Carry ten miR-145-5p target sequences	Target miR-145-5P	[44]
		Ad.DCN	Express decorin protein	Promote the expression of decorin	[45]
		SG400-E2F/IL-15	Replace the endogenous promoter with the E2F-1 promoter and arm with IL-15 gene	Target E2F and promote the expression of IL-15	[46]
Herpes simplex virus	Herpes virus entry mediator (HVEM), nectin1/2, heparan sulfate proteoglycans (HSPG)	G47Δ	Delete α47 and γ34.5 genes; Insert *Escherichia Coli* LacZ into ICP6	Enhance cytotoxicity to tumor cells	[47, 48]
		GD116	Insert MyD116 C-terminus	Enhance the replication and virulence	[49]
		G47Δ-mIL12	Insert IL-12 into G47Δ	Enhance antitumor immune response	[50]
Vaccinia virus	Glycosaminoglycans/ laminin, macrophage receptor with collagenous structure (MARCO)	VV40L	Arm with CD40 ligand	Target CD40	[51]
		VG9-IL-24	Arm with IL-25	Induce apoptosis	[52]
Reovirus	Carbohydrates, Junction adhesion molecule-A(JAM-A)	reo-dox	Conjugate Doxorubicin	Enhance cytotoxicity	[53]
Vesicular Stomatitis Virus	Low-density lipoprotein (LDLR)	VSVd51	A mutation in the matrix protein	Expands the tropism for diverse cancer types	[54]
Measles virus	CD46, nectin-4	rMV-SLAMblind	Mutate R533A in the H protein	Entry cancer cells through nectin-4 receptor	[55]
		rMV-BNiP3	Arm with BNiP3	Enhance the ability of Inducing apoptosis	[56]
Newcastle disease virus	Sialic acid (SA)	rAF-IL12	Insert IL-12	Enhance anti-tumor immune response	[57]
Maraba virus	Low-density lipoprotein receptor (LDLR)	MG1	Mutate G protein (Q242R) and M protein (L123W)	Improve virulence and reduce the ability to kill normal cells	[58, 59]
Coxsackievirus	Coxsackie and adenovirus receptor(CAR), Intracellular adhesion molecule-1(ICAM-1)	CVA21	Natural strain	Capable of lytic infection of breast cancer cells	[60]

#### Adenovirus

Adenoviruses dsDNA viruses are the most studied OVs in breast cancer. Several studies have employed insertion of genes that express antitumor or immune regulatory cytokines in OVs to enhance their effects. IL-24 is an important immune mediator and a cancer suppressor that can specifically inhibit the growth of tumor cells and induce cancer cell apoptosis [61, 62]. Zhu et al. constructed a type 5 adenovirus recombined to carry the IL-24 gene (CNHK600-IL24) that replicates only in tumor cells; they established and treated a TNBC model in nude mice and a metastatic model of breast cancer, concluding that CNHK600-IL24 enhanced antitumor activity and improved survival in vivo [41]. These results clearly demonstrated the potential of adenoviruses to treat TNBC. Furthermore, tropism of OVs on tumor cells can be enhanced by replacing the endogenous promoter with a tumor-specific promoter or enhancer. E2F-1, an important transcriptional factor in the control of cell cycle, proliferation, and carcinogenesis, is often overexpressed in breast cancer tissues compared with normal tissues [63]. Based on this observation, a new oncolytic adenovirus type 5 was recombined by replacing its promoter with E2F-1 promoter and inserting IL-15, an immune regulatory factor; IL-15 is a cytokine that regulates adaptive immune response and inhibits the proliferation of breast cancer cells via various immune cells [64, 65]. Yang et al. confirmed that this recombined OV selectively killed cancer cells while simultaneously releasing IL-15, and enhancing the inhibitory effect on TNBC [46]. Moreover, TRAIL was suggested to induce apoptosis in TNBC with a mesenchymal phenotype [66]. This was tested by constructing modified adenoviruses (P55-HTERT-HRE-TRAIL) harboring TRAIL gene that was subsequently used to infect invasive breast tumor MDA-MB-231, a TNBC cell line [67]. Post-infection, the in vitro adenovirus concentration and expression of TRAIL increased. Further, the decrease in cell viability confirmed its oncolytic potency [42]. Meanwhile, an in vivo orthotopic breast tumor model was established in nude mice; the results of which revealed that the virus significantly restricted tumor growth, thus, corroborating the in vitro results. Additionally, as TNBC has a high metastatic risk, the same study evaluated the anti-metastatic effect of the recombined OV and concluded that P55-HTERT-HRE-TRAIL could restrict TNBC growth and metastasis. Similarly, another study confirmed that green fluorescence protein-expressing telomerase-specific adenovirus (OBP-401) selectively kills cancer cells and has the ability to inhibit TMBC tumor growth and metastasis [43].

#### Herpes simplex virus (HSV)

HSV, another dsDNA OV, is one of the only two OV agents approved for clinical application. Nevertheless, researchers continue to study its application in the treatment of other tumors, including breast cancer. G47Δ, an oncolytic HSV with the US11 promoter region, and deletion of α47, inhibits tumor growth and increases survival rate in vitro in human breast cancer MDA-MB-435 cells, as well as in vivo in a mouse model generated by the implantation of MDA-MB-435 cells or tumor xenografts [68]. Additionally, target genes may be inserted into HSVs to enhance the antitumor effect. In fact, a research team designed a recombined HSV encoding an antitumor cytokine IL-12, G471-mIL12, that efficiently infects and destroys TNBC mammary tumor cells in vitro. In vivo, primary tumor burden and metastasis in the 4 T1 syngeneic TNBC model was significantly reduced at all stages of tumor development [50].

#### Vaccinia virus

Vaccinia virus is a unique dsDNA virus that can replicate in the cell cytoplasm. As an OV, vaccinia virus has great prospective application in the treatment of breast cancer. As IL-24-armed adenovirus exhibited a marked antitumor effect, the vaccinia virus strain Guang9 (VG9) was designed as a vector for carrying IL-24 gene. This recombined virus (VG9-IL-24) kills infected breast cancer cell lines without having any marked cytotoxic effect on normal cells. Next, its antitumor effect was evaluated in vivo by establishing an MDA-MB-231 xenograft mouse model. The mice treated with VG9-IL-24 showed slower tumor progression and longer survival periods with a higher survival rate [52]. As MDA-MB-231 is a TNBC cell line, the success of this research provided evidence for possible application of vaccinia virus in TNBC treatment. In fact, prior to this report, the ability of vaccinia virus to treat TNBC was already established. The GLV-1 h68 oncolytic strain of vaccinia virus reportedly replicates in, and kills, canine mammary tumor cells successfully both in vitro and in vivo (nude mouse model) [69]. Previously, many recombined vaccinia viruses, such as GLV-1 h153 [70] and GLV-1 h164 [71], have been tested in TNBC murine models. Through different artificial transformation methods, their ability to kill TNBC cells in vitro and in TNBC murine models was strengthened, enabling the investigation of their effect and potential application in humans.

#### Reovirus

As a dsRNA OV, reovirus has the potential to be used as a new treatment for breast cancer. A study found that several breast cancer cell lines are susceptible to reovirus infection, while normal breast epithelial cells did not express such characteristic, regardless of hormone receptor status [72]. Moreover, reoviruses can target cancer stem cells (CSCs), which are resistant to conventional clinical treatment. Reoviruses inhibit CSCs and non-CSC cancer cells with equal efficacy, and the sensitivity of CSCs to reovirus therapy is as high as that of non-CSC cells [73]. Based on their killing effect on TNBC cell lines reported in some studies, reoviruses can be considered for potential therapeutic application against TNBC [74]. In addition, reoviruses increase survival and decrease the incidence of leptomeningeal safely and effectively [75]. This evidence indicates that reoviruses have good prospects for treatment of TNBC, which is difficult to treat owing to its high invasion and metastasis ability.

#### Vesicular stomatitis virus (VSV)

VSV is a nonpathogenic negative-sense ssRNA virus. Its characteristic sensitivity to the antiviral activity of type-1 IFNs in normal cells but insensitivity to tumor cells ensures its rapid replication in tumor cells [76]. Symptoms are seen in a very small number of people affected with VSV infections and the pathogenicity of virus is very low and does not cause serious consequences. These advantages make VSV an ideal OV. A recombinant replicating VSV was found to selectively infect, replicate in, and kill breast cancer cells expressing erbb2 [77]. Subsequent studies investigated the ability of VSV to inhibit TNBC. The cytotoxic activity of recombinant VSV (VSVd51) was assessed in mouse and human TNBC cells, where its impact on antitumor immune response was verified. Through the recruitment of NK cells and CD8^+^ T cells, VSV exerted a significant therapeutic effect. This effect on the immune response suggested that VSV in combination with checkpoint inhibitors has the potential to treat TNBC [54].

#### Measles virus (MV)

As early as 2006, reports suggested the potential of MV to treat breast cancer. A study reported that infection with an MV strain, which produces carcinoembryonic antigen (MV-CEA), resulted in MDA-MB-231 cell death in vitro. Further, they tested the antitumor ability of MV-CEA in vivo by establishing subcutaneous MDA-MB-231 xenografts, and concluded that MV-CEA had potent therapeutic efficacy against TNBC both in vitro and in vivo [78]. Further research revealed that these OVs enter cells via the signaling lymphocyte activation molecule (SLAM) expressed on many immune-associated cells, CD46 expressed on all nucleated cells, and the poliovirus receptor-related protein 4 (PVRL4) [79–81]. Based on this, another research group created an MV selectively blind to SLAM (rMV-SLAM blind) that used PVRL4 as a receptor but not CD46 to infect TNBC cells; rMV-SLAM blind was found to decrease the viability of breast cancer cell lines, without affecting the viability of SLAM-positive lymphoid cells. Additionally, rMV-SLAM blind demonstrated greater oncolytic activity than that of wildtype MV as well as its safety [82]. Collectively, these studies provided evidence in support of MV as a candidate for TNBC treatment.

#### Newcastle disease virus (NDV)

NDV belongs to negative-sense ssRNA virus group. The AF2240 strain is one of the most effective oncolytic NDVs that is capable of stimulating apoptosis in breast cancer cells [83, 84]. In TNBC cells, NDV AF2240 can induce tumor regression through upregulation or downregulation of different cytokines, for example, through impairment of IL-6 secretion [84]. To further enhance the antitumor effect, the NDV was artificially modified. Recently, its effect was investigated in breast cancer by recombination with other genes capable of inducing the expression of immune stimulants. In a recent study, inserting IL-2 gene into the NDV genome, was found to induce significant cytotoxicity against MDA-MB-231, while limited cytotoxicity in normal MCF-10A breast cell line. On further testing its antitumor effect in vivo, they concluded, based on observation of tumor size, that this recombined NDV markedly suppressed 4 T1 cells [57].

#### Maraba virus

Maraba virus is a new OVs used in virotherapy and has also been used to treat breast cancer. To verify the possibility of neoadjuvant oncolytic virotherapy, a research team used single direct intratumoral injection of Maraba virus to patient-derived xenografts (PDXs) of TNBC. They concluded that the injection of Maraba virus into 4 T1 tumors effectively initiated virus infection and killing of tumor cells. In subsequent experiments, they developed a rechallenge model to force the reemergence of the disease. They concluded that neoadjuvant oncolytic virotherapy using Maraba virus generated a relatively persistent personalized immune response to kill tumor cells [85]. This study posed a new question about the effective time period for oncolytic virotherapy— ‘is it better to use OV before or after surgery?’ Additionally, other OVs—reovirus, vaccinia virus, and adenovirus—possess the ability to slow tumor growth, while vaccinia virus, HSV, and adenovirus protected against rechallenge and improved survival when administered prior to surgery [86]. These results further illustrated the potential of oncolytic virotherapy as a treatment, even neoadjuvant treatment, for TNBC.

#### Coxsackievirus

Coxsackievirus is an enterovirus with positive-sense ssRNA and has 29 subtypes. Because of its slight pathogenicity to humans, the oncolytic effect of this virus was relatively less studied. However, in upcoming years, genetic engineering can enhance the selectivity of the viruses to tumor cells and reduce their pathogenicity, making the use of coxsackievirus as an OV possible. Coxsackievirus B3 (CV-B3) was genetically engineered to inhibit many tumor cell types, including lung cancer and endometrial cancer [87–89]. Coxsackievirus 21 (CVA21), a genetically unmodified OV, which was shown to be an effective oncolytic agent against multiple myeloma and melanoma [90, 91]. The therapeutic potential of coxsackievirus for TNBC has also been verified. CVA21 could effectively inhibit TNBC cells not only in vitro but also in mouse xenograft models [60]. Although only a few studies have reported the therapeutic effect of coxsackievirus on TNBC, they have demonstrated its potential ability to treat TNBC.

### Research progress of novel OVs in other cancers

In addition to the OVs mentioned above, many others are currently being investigated for their clinical efficacy. Although their successful application for the treatment of TNBC remains to be confirmed, the findings thus far in other malignant tumors suggest that they represent additional potential candidates for TNBC treatment. For instance, poliovirus, which can invade the central nervous system and damage the motor neurons, was found to possess antitumor properties, and suppress the growth of breast cancer in vivo and in vitro using xenografts [92]. The antitumor effect of parvovirus has also been verified in a variety of cancer models, including glioma and pancreatic ductal adenocarcinoma [93, 94]. Similarly, a neuroendocrine cancer-selective oncolytic picornavirus, Seneca Valley virus, exhibits marked inhibitory effects on small cell lung cancer [95]. Zika virus has demonstrated oncolytic activity against glioblastoma stem cells [96, 97]. Meanwhile, the oncolytic parvovirus H-1PV has entered clinical trials targeting glioblastoma and pancreatic cancer [98, 99]. Additionally, a nonhuman pathogen virus, bovine viral diarrhea virus (BVDV), was reported to selectively induce death in human myeloma cell lines (HMCLs), while reducing tumor growth in a mouse model by triggering apoptosis [100]. Another OV, alphavirus M1, which contains a positive-sense single-stranded RNA genome with nonstructural proteins at the 5′ end and structural proteins at the 3′ end, can inhibit the growth of gliomas, as well as hepatic, colorectal, and bladder cancers with no evidence of toxicity or serious adverse events in mice and nonhuman primates [101]. Studies have also indicated that bluetongue virus is capable of infecting and selectively lysing human hepatic carcinoma cells and prostate carcinoma cells, as well as inhibiting human renal cancer cell growth in vitro and in vivo [102–104]. Collectively, these results provide significant support for the further investigation of various OVs for the treatment of TNBC.

### Combination therapy

#### Oncolytic virotherapy combined with radiotherapy and/or chemotherapy

Due to the heterogeneity of cancer, the effect of monotherapy is often unsatisfactory, making combination therapy undoubtedly the best choice for cancer treatment. Meanwhile, the therapeutic potential of OVs can further promote the progress of combination therapy. The effect of such combination therapies have been demonstrated in other cancers as well. For instance, Mori et al. reported a case where a patient with platinum resistant ovarian cancer, with a long history of treatment who was frequently refractory to traditional therapies, showed significant clinical response to combined gl-onc1 OV therapy and chemotherapy [105]. Currently, in addition to surgery, chemotherapy is the most commonly implemented method for the treatment of TNBC. However, combinatorial OV and chemotherapy may elicit synergistic effects, as demonstrated by a team of researchers who combined paclitaxel, a commonly used chemotherapeutic drug, with recombined oncolytic adenovirus to treat gastric cancer in an orthotopic xenograft model. They found that the combination therapy reduced tumor size and strongly enhanced mitotic catastrophe induction due to compulsive cell cycle mobilization of adenoviruses [106]. Moreover, the effect of this combined treatment was superior to that of monotherapy. Additionally, an oncolytic MV armed with BNiP3 (rMV-BNiP3) was combined with paclitaxel to treat TNBC. Compared with either treatment alone, combination therapy induced higher toxicity to TNBC cells, which may have resulted from a significant increase in apoptosis induced by combination therapy [56]. T Similarly, the combination of adenovirus and paclitaxel significantly inhibited the migration and invasion of breast cancer cells [107]. In fact, several studies have reported positive results following administration of various OVs, such as HSV, MV, and reovirus, with other chemotherapeutic drugs, including doxorubicin and camptothecin, to treat TNBC [53, 108, 109].

As radiation may induce killing of OVs, research on the combination of OVs and radiotherapy is limited. Nevertheless, the synergistic antitumor effect of OVs combined with radiotherapy has been confirmed. For instance, Dai et al. observed that oncolytic HSV synergistically enhanced cytolysis in pancreatic cancer cells with radiation via apoptosis, to achieve a therapeutic effect [110]. Moreover, a phase I clinical trial testing the combination of OV, chemotherapy, and radiotherapy reported the delivery of oncolytic vaccinia virus (GL-ONC1) to be safe and feasible in patients with locoregionally advanced head and neck carcinoma [111]. However, the efficacy of OVs combined with radiotherapy for TNBC remains unclear owing to the lack of experimental results. Future research in this direction is required.

#### Oncolytic virotherapy combined with immune checkpoint inhibitors

In addition to chemotherapy and radiotherapy, immune therapy as a new method of cancer treatment has been proposed in recent years. Immune checkpoint (IC) inhibitors (ICIs) showed good therapeutic effects in a variety of cancers [112–115]. One of the reasons for proliferation and metastasis of tumor cells is their ability to escape immune system surveillance, and IC is one of the mechanisms by which they escape immune surveillance [116]. ICs are paired receptor-ligand molecules with interactions that suppress immune responses [117]. The IC receptors programmed death receptor-1 (PD-1) and cytotoxic T lymphocyte antigen 4 are located on the surface of activated T cells and regulatory T cells, respectively [118]]. Binding of receptor and ligand limits T cell activation. Notably, tumor cells always overexpress IC ligands. Hence, tumor cells are not damaged by immune system. ICIs were proposed based on this mechanism and were used in cancer therapy. Immunologists James P. Allison and Tasuku Honjo were awarded the 2018 Nobel Prize in Physiology or Medicine for establishing and proposing negative immunomodulatory therapies for cancer treatment. ICIs block the binding of IC receptors and ligands by binding with them. However, in several cases, the response rate was not ideal. This may be explained by the concept of ‘hot’ and ‘cold’ tumors mentioned previously. It is generally accepted that the effect of ICIs is related to tumor microenvironment, and ICIs can remove the inhibitory effect of tumor cells on T cells to restart the immune response. However, ‘cold’ tumors, which lack immune cells and immune-associated factors in the tumor microenvironment, are free from attack by the immune system. In contrast, ‘hot’ tumor microenvironment can promote the efficacy of ICIs [119, 120]. Thus, the transformation of ‘cold’ tumors into ‘hot’ tumors is an obstacle that needs to be overcome in ICI treatment. As mentioned previously, OVs have the ability to turn ‘cold’ tumor microenvironment into ‘hot’ by releasing TAAs and expressing proinflammatory and immuno-stimulatory cytokines after cancer cell lysis [121]. Additionally, OV infection can promote the secretion of type-1 IFNs that in turn promote PD-L1 expression [122]. Therefore, combined application of OVs with ICIs has been the focus of recent researches. ICIs have always been a hot spot for the treatment of TNBC, and many clinical studies have been performed in this research area [123–126]. Combination therapy with OVs and ICIs provides an effective method to treat TNBC. A study combined CF33-hNIS-ΔF14.5, a chimeric poxvirus, with anti-PD-L1, to test the efficacy of combination therapy. A comparison of the increase in tumor volume and survival of the mice indicated that compared with either anti-PD-L1 or CF33-hNIS-ΔF14.5 treatment alone, the antitumor effect of the combined application was significantly better. Moreover, in tumors treated with CF33-hNIS-ΔF14.5 alone or in combination with anti-PD-L1, generous infiltration of CD8+ T cells was induced. However, anti-PD-L1 treatment alone could not lead to the same result. In the sane experiment, researchers found that the infection of CF33-hNIS-ΔF14.5 upregulated the expression of PD-L1 in human (MDA-MB-468 and Hs578T) and murine (4 T1 and E0771) TNBC cell lines [127]. This may be a possible mechanism via which OVs and ICIs bring about synergistic antitumor effect. As mentioned above, Maraba virus in combination with ICIs could inhibit the growth of TNBC more effectively compared with either treatment alone [85]. Another study reported that the combination of ICIs and alkylating agent temozolomide (TMZ) induced autophagy to increase oncolytic adenovirus replication and oncolysis in TNBC cells in vitro [128].

#### Oncolytic virotherapy combined with chimeric antigen receptor T cell (CAR-T) immunotherapy

In recent years, both OVs and chimeric antigen receptor T cell (CAR-T) therapy have received extensive attention. CAR-T cells are genetically engineered to express specific receptors that recognize specific surface antigens expressed on cell surfaces [129]. As such, CAR-T has been proposed as a useful treatment of tumors. However, although CAR-T therapy has shown promising results in hematologic malignancies, limited efficacy has been noted in solid tumors. Therefore, oncolytic virotherapy in combination with CAR-T therapy was proposed. OVs can be armed with specific target genes to promote the expression of chemokines or cytokines and reverse immunosuppression to promote the migration and survival of CAR-T cells in the TME. For instance, an oncolytic adenovirus armed with the chemokine RANTES and cytokine IL-15 were shown to enhance the survival of CAR-T cells in TME [130]. Similar results were reported in a study that utilized OVs armed with IL-2 and TNF-α [131].

Additionally, by modifying T cells with chimeric antigen receptors that recognize antigens on the surface of cancer cells, CAR-T cells can be designed to specifically target cancer cells [132]. However, some tumors, such as TNBC, lack suitable antigens for specific recognition by CAR-T cells [133], thus, combination with OVs can circumvent this issue. Specifically, OVs can perform targeted delivery of specific genes to tumors, thus, enabling tumor cells to express specific antigens that facilitate CAR-T recognition. Anthony K. Park et.al utilized an oncolytic chimeric orthopoxvirus carrying a CD19t-encoding gene to infect solid tumor cells including those of pancreatic cancer, prostate cancer, and breast cancer. They demonstrated that OVs can effectively deliver the CD19-CAR target to solid tumors and upregulate the expression of CD19t on the surface of these cells. Moreover, OVs promote the CAR-T cells infiltrating tumors to enhance their antitumor effect [134]. Thus, utilizing combined oncolytic virotherapy and CAR-T therapy may represent a major direction for future research on the treatment of solid tumors, specifically in the context of TNBC.

### Impediments in clinical application of OVs

Although modification of OVs, in the studies described here, greatly reduced their toxicity compared with that of wild type viruses, it remains necessary to assess the toxicity of these viruses. An important reason for the use of OVs in cancer treatment is their preference for infecting tumor cells. However, this does not mean that OVs pose zero threat to normal cells. Considering that cancer patients generally receive chemotherapy, radiotherapy, and other treatments that cause immune impairment, there is an inherent risk of serious adverse events developing from OV therapy. Another challenge is the directed delivery of OVs to the tumor site, which is the most critical step in its clinical application. Intravenous infusion is undoubtedly the most convenient and feasible method for drug administration, however, this mode is unsuitable for OV delivery, as they elicit their antitumor effects only after entering tumor cells. Although selective toward tumor cells, OVs are recognized as xenogeneic, thereby inducing immune responses leading to interactions with antigen-presenting cells and resulting in the release of proinflammatory factors [135]. Hence, activation of monocytes and macrophages that phagocytose the virus can prevent it from reaching the tumor site. This antiviral immunity will likely cause OVs to be cleared from the system before reaching the tumor cells. Therefore, improving the efficiency of intravenous infusion should be the primary focus for future research. Meanwhile, although intratumorally injected OVs may be a more suitable route for administration to tumor cells, it is difficult to operate in clinical practice. In addition, intravenous infusion of OVs may be more effective than intratumoral injection [85], because in addition to initiating antiviral immunity, OVs also activate antitumor immunity. Hence, antiviral immunity may stimulate antitumor immunity. Hence, several strategies have been employed to achieve a balance between these two responses.

Coating viral particles with polyethylene glycol, which may prevent their interaction with blood cells, thus, increasing their circulation time in blood stream, was found to reduce transduction of hepatocytes and hepatotoxicity, thereby increasing the anticancer efficacy after systemic (intravenous) administration of OV [136]. Other studies have investigated the use of cell carriers to transport OV as this method may avoid OV elimination during their migration to tumor cells following intravenous injection. In fact, T lymphocytes [137], mesenchymal stem cells [138], and blood outgrowth ECs [139] have been shown to enhance the oncolytic virotherapy as cell carriers. However, these cells do not enhance the effect of OVs after entering the tumor cells. Therefore, further enhancement of the long-term antitumor effect of OVs, as well as their transmission between tumor cells, remains to be achieved.

### Perspectives

Future research must focus on the development of an effective method to reduce the elimination of intravenously infused OVs, thus ensuring that they reach the tumors and replicate. This is essential as the antiviral immunity activated by OVs also suppress the efficacy of oncolytic virotherapy. Furthermore, the timing of OV application has not been thoroughly investigated. Currently, considering that there are no alternatives to surgical interventions, it is necessary to determine if the oncolysis treatment is most efficacious when performed before or after surgery. This is especially important since the replication of OVs requires tumor cells. Thus, OVs may lack a sufficient amount of replication sites post resection of solid tumors, resulting in decreased therapeutic efficiency.

Based on previous clinical data, combination therapy should be the first choice for treatment strategies. Hence, additional data on combination therapies specific for TNBC are needed. Moreover, further clarification of the underlying mechanisms associated with the synergistic effect of combinatorial therapy comprising OVs and radiotherapy or chemotherapy, is required. Moreover, combined treatment need not be limited to two methods. Interestingly, a clinical trial combined the OV, pelareorep, PD-1 inhibitor pembrolizumab, and a chemotherapeutic agent did not induce significant toxicity while showing encouraging efficacy for the treatment of patients with advanced pancreatic adenocarcinoma in this phase Ib study [140]. It is, therefore, necessary to evaluate the efficacy of additional combinations.

Although OVs have shown promising effects in TNBC treatment, based on in vitro and in vivo models, clinical and experimental evidence remains sparce. Nevertheless, clinical trials performed for OVs in other tumors portend the feasibility of their application for TNBC [141]. Hence, in addition to preclinical studies, progress in clinical studies is also essential. Furthermore, in addition to being used in nasopharyngeal carcinoma and melanoma, oncolytic virotherapy is currently used less in clinical settings for other solid tumors, as it remains in the clinical trial (Table 2). As such, there is a lack of clear treatment standards and guidelines for the oncolytic virotherapy of different solid tumors. Finally, it also remains unclear whether treatment options for TNBC and other solid tumors are similar, thus warranting further investigation.

**Table 2:** Clinical trials of OVs

Virus	Disease	Modification	Intervention/treatment	Phase	Number enrolled	dosage-regimen	Primary outcome measure	Secondary outcome measure	Status	NCT number
Adenovirus	Malignant Solid Tumor	Armed with granulocyte-macrophage colony stimulating factor (GMCSF)	CGTG-102	I	12	Intratumoral intravenous	Safety and tolerability; Dose Limiting Toxicities (DLT)	Response rate; disease control rate; progression free and overall survival	Completed	NCT01598129
	Ovarian Cancer; Primary peritoneal cancer	E1A gene deletion. RGD motif binds to integrins avb3 and avb5	Ad5-delta24RGD	I	26	Intraperitoneal	Maximum tolerated dose (MTD); Spectrum of toxicities	Biologic, immunologic and anti-tumor effects of Ad5-delta24RGD	Completed	NCT00562003
	Recurrent platinum resistant ovarian cancer	Developed through a process of bio-selection	Enadenotucirev	I	38	Intravenous	Maximum tolerated dose (MTD)		Completed	NCT02028117
	Pancreatic cancer	Encoding TMZ-CD40L and 4-1BBL(LOAd-703)	LOAd-703; Gemcitabine; nab-paclitaxel; atezolizumab	I/ II	43	Intratumoral	Number of patient with dose-limiting toxicities (DLTs)	Overall Response Rate; Overall Survival	Recruiting	NCT02705196
Reovirus	Metastatic Breast Cancer	No modification	Paclitaxel Reolysin	II	81	Intravenous	Progression free survival	Response rate; Tumor and tissue response of potential molecular factors; Number of patients with adverse events	Completed	NCT01656538
Herpes Simplex Virus	Solid tumor	A spontaneously attenuated mutant	TBI-1401(HF10)	I	6	Intratumoral	Safety and tolerability	Overall tumor response; Levels of antibody to HSV-1	Completed	NCT02428036
	Brain tumors	Deletion of both copies of the γ_**1**_ 34.5 gene and a disabling lacZ insertion in U_L_39 gene (G207)	G207	I	15	Intratumoral	Safety and tolerability	Progression Free Survival; Overall Survival	Recruiting	NCT03911388
	Rhabdomyosarcoma; Osteosarcoma and other	RL1 gene deletion	HSV1716	I	18	Intravenous; Intratumoral	Safety of intratumoral injection or intravenous infusions	Antiviral immune response	Completed	NCT00931931
	Pancreatic cancer	Oncolytic Type 2 Herpes Simplex Virus Expressing Granulocyte Macrophage Colony-Stimulating Factor(OH2)	OH2	I/ II	25	Intratumoral	The objective response rate	Safety; The disease control rate; The duration of response; The progression free survival;	Recruiting	NCT04637698
Vaccinia Virus	Advanced solid tumor	Encoding Renilla luciferase-Aequorea green fluorescent protein fusion, β-galactosidase, and β-glucuronidase	GL-ONC1	I	43	Intravenous	Safety and tolerability	Detection of virus delivery to primary and/or metastatic tumors; Evaluation of anti-vaccinia virus immune response; Determine recommended dose and schedule for future investigation	Completed	NCT00794131
	Hepatocellular Carcinoma (HCC)	Thymidine Kinase deletion; Arm with GM-CSF	JX-594	II	30	Intratumoral	Proportion of Subjects Achieving Disease Control;	Safety and tolerability; Number of Subjects Achieving Disease Control; Median Overall Survival	Completed	NCT00554372
	Hepatocellular Carcinoma (HCC)	Thymidine Kinase deletion; Arm with GM-CSF	Pexastimogene Devacirepvec (Pexa Vec/JX-594) Sorafenib	III	459	Intratumoral	Overall Survival; Overall Response Rate (ORR);	Time to Progression (TTP); Progression Free Survival (PFS); Overall Response Rate (ORR); Disease Control Rate (DCR); Incidence of Adverse Events (AE); Serious Adverse Events (SAE); Time to Symptomatic Progression (TSP)	Completed	NCT02562755
Measles virus	Ovarian cancer	Expressing carcinoembryonic antigen(MV-CEA) Encoding thyroidal sodium iodide symporter (MV-NIS)	MV-CEA, MV-NIS	I	37	Intraperitoneally	Dose limiting Toxicity (DLT); Maximum tolerated dose (MTD)	Number of Responses; CA-125 levels; Time to Progression	Completed	NCT00408590

## Conclusions

OVs can be considered potential candidates for the treatment of TNBC owing to their abilities to replicate in tumor cells, causing their direct lysis, destroy tumor vasculature, activate innate and adaptive immune responses to generate an antitumor effect, transform ‘cold’ tumors to ‘hot’, act as a vector carrying target genes and expressing antitumor related factors in tumor cells. These characteristics make OVs advantageous, compared with other treatment modalities, including radiotherapy, chemotherapy, and endocrine therapy. Moreover, their preference for tumor cells reduces the adverse events elicited by OVs to normal cells, while also reducing the development of drug resistance. Moreover, as the viruses actively replicate following entry into host cells, they effectively activate the immune system, thus, maintaining a long-term antitumor effect. Hence, to address the urgent requirement for advanced and novel treatment strategies for TNBC, the feasibility of OVs must continue to be investigated as a means of opening new avenues of research and providing a novel platform for clinical treatment.

## Data Availability

Not applicable.

## Abbreviations

CSC: Cancer stem cells
DAMP: Danger-associated molecular patterns
ER: Estrogen receptor
HSV: Herpes simplex virus
IC: Immune checkpoint
MV: Measles virus
NDV: Newcastle disease virus
NK: Natural killer
OV: Oncolytic virus
PAMP: Pathogen-associated molecular patterns
PDX: Patient-derived xenografts
PR: Progesterone receptor
PRR: Pattern recognition receptors
SLAM: Signaling lymphocyte activation molecule
TAA: Tumor-associated antigens
TNBC: Triple negative breast cancer
TRAIL: TNF-related apoptosis inducing ligand
VSV: Vesicular stomatitis virus

## References

[1] Russell SJ, Peng KW (2007). Viruses as anticancer drugs. Trends Pharmacol Sci.

[2] Gujar S, Pol JG, Kim Y, Lee PW, Kroemer G (2018). Antitumor benefits of antiviral immunity: an underappreciated aspect of oncolytic virotherapies. Trends Immunol.

[3] Kelly E, Russell SJ (2007). History of oncolytic viruses: genesis to genetic engineering. Mol Ther.

[4] Huebner RJ, Rowe WP, Schatten WE, Smith RR, Thomas LB (1956). Studies on the use of viruses in the treatment of carcinoma of the cervix. Cancer..

[5] Watanabe D, Goshima F (2018). Oncolytic Virotherapy by HSV. Adv Exp Med Biol.

[6] Jeong SN, Yoo SY (2020). Novel oncolytic virus armed with cancer suicide gene and normal vasculogenic gene for improved anti-tumor activity. Cancers (Basel).

[7] Ricordel M, Foloppe J, Pichon C, Sfrontato N, Antoine D, Tosch C (2017). Cowpox virus: a new and armed oncolytic poxvirus. Mol Ther Oncolytics.

[8] Mathis JM, SM OB (2018). Oncolytic virotherapy for breast cancer treatment. Curr Gene Ther.

[9] Bray F, Ferlay J, Soerjomataram I, Siegel RL, Torre LA, Jemal A (2018). Global cancer statistics 2018: GLOBOCAN estimates of incidence and mortality worldwide for 36 cancers in 185 countries. CA Cancer J Clin.

[10] Subik K, Lee JF, Baxter L, Strzepek T, Costello D, Crowley P, Xing L, Hung MC, Bonfiglio T, Hicks DG, Tang P (2010). The expression patterns of ER, PR, HER2, CK5/6, EGFR, Ki-67 and AR by Immunohistochemical analysis in breast cancer cell lines. Breast Cancer (Auckl).

[11] Rakha EA, El-Sayed ME, Green AR, Lee AH, Robertson JF, Ellis IO (2007). Prognostic markers in triple-negative breast cancer. Cancer..

[12] Abramson VG, Lehmann BD, Ballinger TJ, Pietenpol JA (2015). Subtyping of triple-negative breast cancer: implications for therapy. Cancer..

[13] Dent R, Trudeau M, Pritchard KI, Hanna WM, Kahn HK, Sawka CA, Lickley LA, Rawlinson E, Sun P, Narod SA (2007). Triple-negative breast cancer: clinical features and patterns of recurrence. Clin Cancer Res.

[14] Smid M, Wang Y, Zhang Y, Sieuwerts AM, Yu J, Klijn JG (2008). Subtypes of breast cancer show preferential site of relapse. Cancer Res.

[15] Anders CK, Carey LA (2009). Biology, metastatic patterns, and treatment of patients with triple-negative breast cancer. Clin Breast Cancer.

[16] Kennedy CR, Gao F, Margenthaler JA (2010). Neoadjuvant versus adjuvant chemotherapy for triple negative breast cancer. J Surg Res.

[17] Park JH, Ahn JH, Kim SB (2018). How shall we treat early triple-negative breast cancer (TNBC): from the current standard to upcoming immuno-molecular strategies. ESMO Open.

[18] Bai X, Ni J, Beretov J, Graham P, Li Y (2021). Triple-negative breast cancer therapeutic resistance: where is the Achilles’ heel?. Cancer Lett.

[19] Wei D, Xu J, Liu XY, Chen ZN, Bian H (2018). Fighting cancer with viruses: oncolytic virus therapy in China. Hum Gene Ther.

[20] Printz C (2016). FDA approves new treatment of metastatic melanoma. Cancer..

[21] Santry LA, van Vloten JP, Knapp JP, Matuszewska K, McAusland TM, Minott JA (2020). Tumour vasculature: friend or foe of oncolytic viruses?. Cytokine Growth Factor Rev.

[22] Zhang Y, Li Y, Chen K, Qian L, Wang P (2021). Oncolytic virotherapy reverses the immunosuppressive tumor microenvironment and its potential in combination with immunotherapy. Cancer Cell Int.

[23] Benencia F, Courreges MC, Conejo-Garcia JR, Buckanovich RJ, Zhang L, Carroll RH (2005). Oncolytic HSV exerts direct antiangiogenic activity in ovarian carcinoma. Hum Gene Ther.

[24] Gujar S, Bell J, Diallo JS (2019). SnapShot: cancer immunotherapy with oncolytic viruses. Cell..

[25] Breitbach CJ, De Silva NS, Falls TJ, Aladl U, Evgin L, Paterson J (2011). Targeting tumor vasculature with an oncolytic virus. Mol Ther.

[26] Breitbach CJ, Paterson JM, Lemay CG, Falls TJ, McGuire A, Parato KA, Stojdl DF, Daneshmand M, Speth K, Kirn D, McCart JA, Atkins H, Bell JC (2007). Targeted inflammation during oncolytic virus therapy severely compromises tumor blood flow. Mol Ther.

[27] Souza-Fonseca-Guimaraes F, Cursons J, Huntington ND (2019). The emergence of natural killer cells as a major target in cancer immunotherapy. Trends Immunol.

[28] Di Vito C, Mikulak J, Zaghi E, Pesce S, Marcenaro E, Mavilio D (2019). NK cells to cure cancer. Semin Immunol.

[29] Andzinski L, Kasnitz N, Stahnke S, Wu CF, Gereke M, von Kockritz-Blickwede M (2016). Type I IFNs induce anti-tumor polarization of tumor associated neutrophils in mice and human. Int J Cancer.

[30] Melchjorsen J (2013). Learning from the messengers: innate sensing of viruses and cytokine regulation of immunity - clues for treatments and vaccines. Viruses..

[31] Stanford MM, Breitbach CJ, Bell JC, McFadden G (2008). Innate immunity, tumor microenvironment and oncolytic virus therapy: friends or foes?. Curr Opin Mol Ther.

[32] Krysko DV, Garg AD, Kaczmarek A, Krysko O, Agostinis P, Vandenabeele P (2012). Immunogenic cell death and DAMPs in cancer therapy. Nat Rev Cancer.

[33] Kroemer G, Galluzzi L, Kepp O, Zitvogel L (2013). Immunogenic cell death in cancer therapy. Annu Rev Immunol.

[34] Kim Y, Clements DR, Sterea AM, Jang HW, Gujar SA, Lee PW (2015). Dendritic cells in oncolytic virus-based anti-cancer therapy. Viruses..

[35] Steinman RM, Hemmi H (2006). Dendritic cells: translating innate to adaptive immunity. Curr Top Microbiol Immunol.

[36] Marchini A, Daeffler L, Pozdeev VI, Angelova A, Rommelaere J (2019). Immune conversion of tumor microenvironment by oncolytic viruses: the protoparvovirus H-1PV case study. Front Immunol.

[37] Galon J, Bruni D (2019). Approaches to treat immune hot, altered and cold tumours with combination immunotherapies. Nat Rev Drug Discov.

[38] Bai Y, Hui P, Du X, Su X (2019). Updates to the antitumor mechanism of oncolytic virus. Thorac Cancer.

[39] Pidelaserra-Marti G, Engeland CE (2020). Mechanisms of measles virus oncolytic immunotherapy. Cytokine Growth Factor Rev.

[40] Patel MR, Jacobson BA, Ji Y, Drees J, Tang S, Xiong K, Wang H, Prigge JE, Dash AS, Kratzke AK, Mesev E, Etchison R, Federspiel MJ, Russell SJ, Kratzke RA (2015). Vesicular stomatitis virus expressing interferon-beta is oncolytic and promotes antitumor immune responses in a syngeneic murine model of non-small cell lung cancer. Oncotarget..

[41] Zhu W, Wei L, Zhang H, Chen J, Qin X (2012). Oncolytic adenovirus armed with IL-24 inhibits the growth of breast cancer in vitro and in vivo. J Exp Clin Cancer Res.

[42] Zhu W, Zhang H, Shi Y, Song M, Zhu B, Wei L (2013). Oncolytic adenovirus encoding tumor necrosis factor-related apoptosis inducing ligand (TRAIL) inhibits the growth and metastasis of triple-negative breast cancer. Cancer Biol Ther.

[43] Yano S, Takehara K, Kishimoto H, Tazawa H, Urata Y, Kagawa S, Bouvet M, Fujiwara T, Hoffman RM (2016). Tumor-targeting adenovirus OBP-401 inhibits primary and metastatic tumor growth of triple-negative breast cancer in orthotopic nude-mouse models. Oncotarget..

[44] Shayestehpour M, Moghim S, Salimi V, Jalilvand S, Yavarian J, Romani B (2017). Targeting human breast cancer cells by an oncolytic adenovirus using microRNA-targeting strategy. Virus Res.

[45] Zhao H, Wang H, Kong F, Xu W, Wang T, Xiao F, Wang L, Huang D, Seth P, Yang Y, Wang H (2019). Oncolytic adenovirus rAd.DCN inhibits breast tumor growth and lung metastasis in an immune-competent orthotopic xenograft model. Hum Gene Ther.

[46] Yan Y, Xu H, Wang J, Wu X, Wen W, Liang Y (2019). Inhibition of breast cancer cells by targeting E2F-1 gene and expressing IL15 oncolytic adenovirus. Biosci Rep.

[47] Wang J, Hu P, Zeng M, Rabkin SD, Liu R (2012). Oncolytic herpes simplex virus treatment of metastatic breast cancer. Int J Oncol.

[48] Peters C, Rabkin SD (2015). Designing herpes viruses as oncolytics. Mol Ther Oncolytics.

[49] Cheng L, Jiang H, Fan J, Wang J, Hu P, Ruan Y, Liu R (2018). A novel oncolytic herpes simplex virus armed with the carboxyl-terminus of murine MyD116 has enhanced anti-tumour efficacy against human breast cancer cells. Oncol Lett.

[50] Ghouse SM, Nguyen HM, Bommareddy PK, Guz-Montgomery K, Saha D (2020). Oncolytic herpes simplex virus encoding IL12 controls triple-negative breast cancer growth and metastasis. Front Oncol.

[51] Governa V, Brittoli A, Mele V, Pinamonti M, Terracciano L, Muenst S, Iezzi G, Spagnoli GC, Zajac P, Trella E (2019). A replication-incompetent CD154/40L recombinant vaccinia virus induces direct and macrophage-mediated antitumor effects in vitro and in vivo. Oncoimmunology..

[52] Deng L, Fan J, Ding Y, Yang X, Huang B, Hu Z (2020). Target therapy with vaccinia virus harboring IL-24 for human breast cancer. J Cancer.

[53] Berry JTL, Munoz LE, Rodriguez Stewart RM, Selvaraj P, Mainou BA (2020). Doxorubicin conjugation to reovirus improves oncolytic efficacy in triple-negative breast cancer. Mol Ther Oncolytics.

[54] Niavarani SR, Lawson C, Boudaud M, Simard C, Tai LH (2020). Oncolytic vesicular stomatitis virus-based cellular vaccine improves triple-negative breast cancer outcome by enhancing natural killer and CD8(+) T-cell functionality. J Immunother Cancer.

[55] Fujiyuki T, Amagai Y, Shoji K, Kuraishi T, Sugai A, Awano M, Sato H, Hattori S, Yoneda M, Kai C (2020). Recombinant SLAMblind measles virus is a promising candidate for nectin-4-positive triple negative breast cancer therapy. Mol Ther Oncolytics.

[56] Lal G, Rajala MS (2018). Combination of oncolytic measles virus armed with BNiP3, a pro-apoptotic gene and paclitaxel induces breast cancer cell death. Front Oncol.

[57] Mohamed Amin Z, Che Ani MA, Tan SW, Yeap SK, Alitheen NB, Syed Najmuddin SUF, Kalyanasundram J, Chan SC, Veerakumarasivam A, Chia SL, Yusoff K (2019). Evaluation of a recombinant Newcastle disease virus expressing human IL12 against human breast cancer. Sci Rep.

[58] Brun J, McManus D, Lefebvre C, Hu K, Falls T, Atkins H, Bell JC, McCart JA, Mahoney D, Stojdl DF (2010). Identification of genetically modified Maraba virus as an oncolytic rhabdovirus. Mol Ther.

[59] Bourgeois-Daigneault MC, St-Germain LE, Roy DG, Pelin A, Aitken AS, Arulanandam R, Falls T, Garcia V, Diallo JS, Bell JC (2016). Combination of paclitaxel and MG1 oncolytic virus as a successful strategy for breast cancer treatment. Breast Cancer Res.

[60] Skelding KA, Barry RD, Shafren DR (2009). Systemic targeting of metastatic human breast tumor xenografts by Coxsackievirus A21. Breast Cancer Res Treat.

[61] Kreis S, Philippidou D, Margue C, Behrmann I (2008). IL-24: a classic cytokine and/or a potential cure for cancer?. J Cell Mol Med.

[62] Menezes ME, Bhatia S, Bhoopathi P, Das SK, Emdad L, Dasgupta S (2014). MDA-7/IL-24: multifunctional cancer killing cytokine. Adv Exp Med Biol.

[63] Johnson J, Thijssen B, McDermott U, Garnett M, Wessels LF, Bernards R (2016). Targeting the RB-E2F pathway in breast cancer. Oncogene..

[64] Heon EK, Wulan H, Macdonald LP, Malek AO, Braunstein GH, Eaves CG, Schattner MD, Allen PM, Alexander MO, Hawkins CA, McGovern DW, Freeman RL, Amir EP, Huse JD, Zaltzman JS, Kauff NP, Meyers PG, Gleason MH, Overholtzer MG, Wiseman SS, Streutker CD, Asa SW, McAlindon TP, Newcomb PO, Sorensen PM, Press OA (2015). IL-15 induces strong but short-lived tumor-infiltrating CD8 T cell responses through the regulation of Tim-3 in breast cancer. Biochem Biophys Res Commun.

[65] Gillgrass A, Gill N, Babian A, Ashkar AA (2014). The absence or overexpression of IL-15 drastically alters breast cancer metastasis via effects on NK cells, CD4 T cells, and macrophages. J Immunol.

[66] Rahman M, Davis SR, Pumphrey JG, Bao J, Nau MM, Meltzer PS, Lipkowitz S (2009). TRAIL induces apoptosis in triple-negative breast cancer cells with a mesenchymal phenotype. Breast Cancer Res Treat.

[67] Neve RM, Chin K, Fridlyand J, Yeh J, Baehner FL, Fevr T, Clark L, Bayani N, Coppe JP, Tong F, Speed T, Spellman PT, DeVries S, Lapuk A, Wang NJ, Kuo WL, Stilwell JL, Pinkel D, Albertson DG, Waldman FM, McCormick F, Dickson RB, Johnson MD, Lippman M, Ethier S, Gazdar A, Gray JW (2006). A collection of breast cancer cell lines for the study of functionally distinct cancer subtypes. Cancer Cell.

[68] Liu RB, Rabkin SD (2005). Oncolytic herpes simplex virus vectors for the treatment of human breast cancer. Chin Med J.

[69] Gentschev I, Stritzker J, Hofmann E, Weibel S, Yu YA, Chen N, Zhang Q, Bullerdiek J, Nolte I, Szalay AA (2009). Use of an oncolytic vaccinia virus for the treatment of canine breast cancer in nude mice: preclinical development of a therapeutic agent. Cancer Gene Ther.

[70] Gholami S, Chen CH, Belin LJ, Lou E, Fujisawa S, Antonacci C, Carew A, Chen NG, de Brot M, Zanzonico PB, Szalay AA, Fong Y (2013). Vaccinia virus GLV-1h153 is a novel agent for detection and effective local control of positive surgical margins for breast cancer. Breast Cancer Res.

[71] Gholami S, Marano A, Chen NG, Aguilar RJ, Frentzen A, Chen CH, Lou E, Fujisawa S, Eveno C, Belin L, Zanzonico P, Szalay A, Fong Y (2014). A novel vaccinia virus with dual oncolytic and anti-angiogenic therapeutic effects against triple-negative breast cancer. Breast Cancer Res Treat.

[72] Hata Y, Etoh T, Inomata M, Shiraishi N, Nishizono A, Kitano S (2008). Efficacy of oncolytic reovirus against human breast cancer cells. Oncol Rep.

[73] Marcato P, Dean CA, Giacomantonio CA, Lee PW (2009). Oncolytic reovirus effectively targets breast cancer stem cells. Mol Ther.

[74] Norman KL, Coffey MC, Hirasawa K, Demetrick DJ, Nishikawa SG, DiFrancesco LM (2002). Reovirus oncolysis of human breast cancer. Hum Gene Ther.

[75] Yang WQ, Senger DL, Lun XQ, Muzik H, Shi ZQ, Dyck RH, Norman K, Brasher PMA, Rewcastle NB, George D, Stewart D, Lee PWK, Forsyth PA (2004). Reovirus as an experimental therapeutic for brain and leptomeningeal metastases from breast cancer. Gene Ther.

[76] Balachandran S, Barber GN (2000). Vesicular stomatitis virus (VSV) therapy of tumors. IUBMB Life.

[77] Bergman I, Whitaker-Dowling P, Gao Y, Griffin JA (2004). Preferential targeting of vesicular stomatitis virus to breast cancer cells. Virology..

[78] McDonald CJ, Erlichman C, Ingle JN, Rosales GA, Allen C, Greiner SM (2006). A measles virus vaccine strain derivative as a novel oncolytic agent against breast cancer. Breast Cancer Res Treat.

[79] Tatsuo H, Ono N, Tanaka K, Yanagi Y (2000). SLAM (CDw150) is a cellular receptor for measles virus. Nature..

[80] Dorig RE, Marcil A, Chopra A, Richardson CD (1993). The human CD46 molecule is a receptor for measles virus (Edmonston strain). Cell..

[81] Muhlebach MD, Mateo M, Sinn PL, Prufer S, Uhlig KM, Leonard VH (2011). Adherens junction protein nectin-4 is the epithelial receptor for measles virus. Nature..

[82] Sugiyama T, Yoneda M, Kuraishi T, Hattori S, Inoue Y, Sato H, Kai C (2013). Measles virus selectively blind to signaling lymphocyte activation molecule as a novel oncolytic virus for breast cancer treatment. Gene Ther.

[83] Alabsi AM, Ali R, Ideris A, Omar AR, Bejo MH, Yusoff K (2012). Anti-leukemic activity of Newcastle disease virus strains AF2240 and V4-UPM in murine myelomonocytic leukemia in vivo. Leuk Res.

[84] Raihan J, Ahmad U, Yong YK, Eshak Z, Othman F, Ideris A (2019). Regression of solid breast tumours in mice by Newcastle disease virus is associated with production of apoptosis related-cytokines. BMC Cancer.

[85] Bourgeois-Daigneault MC, Roy DG, Aitken AS, El Sayes N, Martin NT, Varette O (2018). Neoadjuvant oncolytic virotherapy before surgery sensitizes triple-negative breast cancer to immune checkpoint therapy. Sci Transl Med.

[86] Martin NT, Roy DG, Workenhe ST, van den Wollenberg DJM, Hoeben RC, Mossman KL, Bell JC, Bourgeois-Daigneault MC (2019). Pre-surgical neoadjuvant oncolytic virotherapy confers protection against rechallenge in a murine model of breast cancer. Sci Rep.

[87] Lin Y, Wang W, Wan J, Yang Y, Fu W, Pan D, Cai L, Cheng T, Huang X, Wang Y (2018). Oncolytic activity of a coxsackievirus B3 strain in human endometrial cancer cell lines. Virol J.

[88] Miyamoto S, Inoue H, Nakamura T, Yamada M, Sakamoto C, Urata Y, Okazaki T, Marumoto T, Takahashi A, Takayama K, Nakanishi Y, Shimizu H, Tani K (2012). Coxsackievirus B3 is an oncolytic virus with immunostimulatory properties that is active against lung adenocarcinoma. Cancer Res.

[89] Cai L, Liu Z (2020). Novel recombinant coxsackievirus B3 with genetically inserted basic peptide elicits robust antitumor activity against lung cancer. Cancer Med.

[90] Au GG, Lincz LF, Enno A, Shafren DR (2007). Oncolytic Coxsackievirus A21 as a novel therapy for multiple myeloma. Br J Haematol.

[91] Shafren DR, Au GG, Nguyen T, Newcombe NG, Haley ES, Beagley L (2004). Systemic therapy of malignant human melanoma tumors by a common cold-producing enterovirus, coxsackievirus a21. Clin Cancer Res.

[92] Holl EK, Brown MC, Boczkowski D, McNamara MA, George DJ, Bigner DD (2016). Recombinant oncolytic poliovirus, PVSRIPO, has potent cytotoxic and innate inflammatory effects, mediating therapy in human breast and prostate cancer xenograft models. Oncotarget..

[93] Angelova A, Rommelaere J (2019). Immune system stimulation by oncolytic rodent protoparvoviruses. Viruses.

[94] Bhat R, Dempe S, Dinsart C, Rommelaere J (2011). Enhancement of NK cell antitumor responses using an oncolytic parvovirus. Int J Cancer.

[95] Poirier JT, Dobromilskaya I, Moriarty WF, Peacock CD, Hann CL, Rudin CM (2013). Selective tropism of Seneca Valley virus for variant subtype small cell lung cancer. J Natl Cancer Inst.

[96] Zhu Z, Gorman MJ, McKenzie LD, Chai JN, Hubert CG, Prager BC (2017). Zika virus has oncolytic activity against glioblastoma stem cells. J Exp Med.

[97] Lubin JA, Zhang RR, Kuo JS (2018). Zika virus has oncolytic activity against glioblastoma stem cells. Neurosurgery..

[98] Geletneky K, Hajda J, Angelova AL, Leuchs B, Capper D, Bartsch AJ (2017). Oncolytic H-1 parvovirus shows safety and signs of immunogenic activity in a first phase I/IIa glioblastoma trial. Mol Ther.

[99] Hajda J, Lehmann M, Krebs O, Kieser M, Geletneky K, Jager D (2017). A non-controlled, single arm, open label, phase II study of intravenous and intratumoral administration of ParvOryx in patients with metastatic, inoperable pancreatic cancer: ParvOryx02 protocol. BMC Cancer.

[100] Marchica V, Franceschi V, Vescovini R, Storti P, Vicario E, Toscani D, Zorzoli A, Airoldi I, Dalla Palma B, Campanini N, Martella E, Mancini C, Costa F, Donofrio G, Giuliani N (2020). Bovine pestivirus is a new alternative virus for multiple myeloma oncolytic virotherapy. J Hematol Oncol.

[101] Muhuri M, Gao G (2021). Oncolytic virus alphavirus M1: a new and promising weapon to fight cancer. Hum Gene Ther.

[102] Hu J, Dong CY, Li JK, Chen DE, Liang K, Liu J (2008). Selective in vitro cytotoxic effect of human cancer cells by bluetongue virus-10. Acta Oncol.

[103] Wang W, Chen MN, Cheng K, Zhan LL, Zhang J (2014). Cytotoxic effect of a combination of bluetongue virus and radiation on prostate cancer. Exp Ther Med.

[104] Wang H, Song L, Zhang X, Zhang X, Zhou X (2021). Bluetongue viruses act as novel oncolytic viruses to effectively inhibit human renal cancer cell growth in vitro and in vivo. Med Sci Monit.

[105] Mori KM, Giuliano PD, Lopez KL, King MM, Bohart R, Goldstein BH (2019). Pronounced clinical response following the oncolytic vaccinia virus GL-ONC1 and chemotherapy in a heavily pretreated ovarian cancer patient. Anti-Cancer Drugs.

[106] Ishikawa W, Kikuchi S, Ogawa T, Tabuchi M, Tazawa H, Kuroda S, Noma K, Nishizaki M, Kagawa S, Urata Y, Fujiwara T (2020). Boosting replication and penetration of oncolytic adenovirus by paclitaxel eradicate peritoneal metastasis of gastric cancer. Mol Ther Oncolytics.

[107] Wang J, Li Y, Li S, Yao W, Liu X, Zhu Y, Li W, Sun L, Jin N, Li X (2020). Anti-tumor synergistic effect of a dual cancer-specific recombinant adenovirus and paclitaxel on breast cancer. Front Oncol.

[108] Zeng WG, Li JJ, Hu P, Lei L, Wang JN, Liu RB (2013). An oncolytic herpes simplex virus vector, G47Delta, synergizes with paclitaxel in the treatment of breast cancer. Oncol Rep.

[109] Tai CJ, Liu CH, Pan YC, Wong SH, Tai CJ, Richardson CD, Lin LT (2019). Chemovirotherapeutic treatment using camptothecin enhances oncolytic measles virus-mediated killing of breast cancer cells. Sci Rep.

[110] Dai MH, Zamarin D, Gao SP, Chou TC, Gonzalez L, Lin SF, Fong Y (2010). Synergistic action of oncolytic herpes simplex virus and radiotherapy in pancreatic cancer cell lines. Br J Surg.

[111] Mell LK, Brumund KT, Daniels GA, Advani SJ, Zakeri K, Wright ME (2017). Phase I trial of intravenous oncolytic vaccinia virus (GL-ONC1) with cisplatin and radiotherapy in patients with locoregionally advanced head and neck carcinoma. Clin Cancer Res.

[112] Johansson H, Andersson R, Bauden M, Hammes S, Holdenrieder S, Ansari D (2016). Immune checkpoint therapy for pancreatic cancer. World J Gastroenterol.

[113] Kim BJ, Jang HJ, Kim HS, Kim JH (2017). Current status of immune checkpoint inhibitors in gastrointestinal cancers. J Cancer.

[114] Byrne EH, Fisher DE (2017). Immune and molecular correlates in melanoma treated with immune checkpoint blockade. Cancer..

[115] Madden K, Kasler MK (2019). Immune checkpoint inhibitors in lung cancer and melanoma. Semin Oncol Nurs.

[116] Beatty GL, Gladney WL (2015). Immune escape mechanisms as a guide for cancer immunotherapy. Clin Cancer Res.

[117] Topalian SL (2017). Targeting immune checkpoints in Cancer therapy. JAMA..

[118] Sivanandam V, LaRocca CJ, Chen NG, Fong Y, Warner SG (2019). Oncolytic viruses and immune checkpoint inhibition: the best of both worlds. Mol Ther Oncolytics.

[119] Sharma P, Hu-Lieskovan S, Wargo JA, Ribas A (2017). Primary, adaptive, and acquired resistance to cancer immunotherapy. Cell..

[120] Bonaventura P, Shekarian T, Alcazer V, Valladeau-Guilemond J, Valsesia-Wittmann S, Amigorena S, Caux C, Depil S (2019). Cold tumors: a therapeutic challenge for immunotherapy. Front Immunol.

[121] Gujar S, Pol JG, Kroemer G (2018). Heating it up: oncolytic viruses make tumors ‘hot’ and suitable for checkpoint blockade immunotherapies. Oncoimmunology..

[122] Terawaki S, Chikuma S, Shibayama S, Hayashi T, Yoshida T, Okazaki T, Honjo T (2011). IFN-alpha directly promotes programmed cell death-1 transcription and limits the duration of T cell-mediated immunity. J Immunol.

[123] Nanda R, Chow LQ, Dees EC, Berger R, Gupta S, Geva R (2016). Pembrolizumab in patients with advanced triple-negative breast cancer: phase Ib KEYNOTE-012 study. J Clin Oncol.

[124] Adams S, Loi S, Toppmeyer D, Cescon DW, De Laurentiis M, Nanda R (2019). Pembrolizumab monotherapy for previously untreated, PD-L1-positive, metastatic triple-negative breast cancer: cohort B of the phase II KEYNOTE-086 study. Ann Oncol.

[125] Emens LA, Cruz C, Eder JP, Braiteh F, Chung C, Tolaney SM (2019). Long-term clinical outcomes and biomarker analyses of atezolizumab therapy for patients with metastatic triple-negative breast cancer: a phase 1 study. JAMA Oncol.

[126] Adams S, Schmid P, Rugo HS, Winer EP, Loirat D, Awada A, Cescon DW, Iwata H, Campone M, Nanda R, Hui R, Curigliano G, Toppmeyer D, O’Shaughnessy J, Loi S, Paluch-Shimon S, Tan AR, Card D, Zhao J, Karantza V, Cortés J (2019). Pembrolizumab monotherapy for previously treated metastatic triple-negative breast cancer: cohort A of the phase II KEYNOTE-086 study. Ann Oncol.

[127] Chaurasiya S, Yang A, Kang S, Lu J, Kim SI, Park AK (2020). Oncolytic poxvirus CF33-hNIS-DeltaF14.5 favorably modulates tumor immune microenvironment and works synergistically with anti-PD-L1 antibody in a triple-negative breast cancer model. Oncoimmunology.

[128] Garza-Morales R, Gonzalez-Ramos R, Chiba A, Montes de Oca-Luna R, McNally LR, McMasters KM (2018). Temozolomide enhances triple-negative breast cancer virotherapy in vitro. Cancers (Basel).

[129] Marofi F, Motavalli R, Safonov VA, Thangavelu L, Yumashev AV, Alexander M, Shomali N, Chartrand MS, Pathak Y, Jarahian M, Izadi S, Hassanzadeh A, Shirafkan N, Tahmasebi S, Khiavi FM (2021). CAR T cells in solid tumors: challenges and opportunities. Stem Cell Res Ther.

[130] Nishio N, Diaconu I, Liu H, Cerullo V, Caruana I, Hoyos V, Bouchier-Hayes L, Savoldo B, Dotti G (2014). Armed oncolytic virus enhances immune functions of chimeric antigen receptor-modified T cells in solid tumors. Cancer Res.

[131] Watanabe K, Luo Y, Da T, Guedan S, Ruella M, Scholler J (2018). Pancreatic cancer therapy with combined mesothelin-redirected chimeric antigen receptor T cells and cytokine-armed oncolytic adenoviruses. JCI Insight.

[132] Ajina A, Maher J (2017). Prospects for combined use of oncolytic viruses and CAR T-cells. J Immunother Cancer.

[133] Schmidts A, Maus MV (2018). Making CAR T cells a solid option for solid tumors. Front Immunol.

[134] Park AK, Fong Y, Kim SI, Yang J, Murad JP, Lu J (2020). Effective combination immunotherapy using oncolytic viruses to deliver CAR targets to solid tumors. Sci Transl Med.

[135] Hagedorn C, Kreppel F (2017). Capsid engineering of adenovirus vectors: overcoming early vector-host interactions for therapy. Hum Gene Ther.

[136] Doronin K, Shashkova EV, May SM, Hofherr SE, Barry MA (2009). Chemical modification with high molecular weight polyethylene glycol reduces transduction of hepatocytes and increases efficacy of intravenously delivered oncolytic adenovirus. Hum Gene Ther.

[137] Ong HT, Hasegawa K, Dietz AB, Russell SJ, Peng KW (2007). Evaluation of T cells as carriers for systemic measles virotherapy in the presence of antiviral antibodies. Gene Ther.

[138] Ramirez M, Garcia-Castro J, Melen GJ, Gonzalez-Murillo A, Franco-Luzon L (2015). Patient-derived mesenchymal stem cells as delivery vehicles for oncolytic virotherapy: novel state-of-the-art technology. Oncolytic Virother.

[139] Patel MR, Jacobson BA, Ji Y, Hebbel RP, Kratzke RA (2020). Blood outgrowth endothelial cells as a cellular carrier for oncolytic vesicular stomatitis virus expressing interferon-beta in preclinical models of non-small cell lung cancer. Transl Oncol.

[140] Mahalingam D, Wilkinson GA, Eng KH, Fields P, Raber P, Moseley JL (2020). Pembrolizumab in combination with the oncolytic virus pelareorep and chemotherapy in patients with advanced pancreatic adenocarcinoma: a phase Ib study. Clin Cancer Res.

[141] Macedo N, Miller DM, Haq R, Kaufman HL (2020). Clinical landscape of oncolytic virus research in 2020. J Immunother Cancer.

